# Treatment of Scurvy

**Published:** 1883-02

**Authors:** 


### Treatment of Scurvy.

A correspondent writes to the *British Medical Journal* reporting the following case:

A young man with spinal disease had all the signs of sea-scurvy, spongy gums, etc. I ascertained that vegetables had been repugnant to him, and for a long period he had refrained from taking any. The use of vegetables and turpentine, with potash, both cheap remedies, wrought a speedy cure. I beg leave to suggest these drugs upon an extensive scale, as on shipboard. I believe alkalies more effective when not given, for internal or external use, in a state of chemical combination likely to neutralize the effect, *e. g.*, the citrates internally, or sulphur with lime externally, in the treatment of scabies. I prefer free sulphur, and it is the most reliable.

